# A bee’s-eye view of landscape change: differences in diet of 2 *Andrena* species (Hymenoptera: Andrenidae) between 1943 and 2021

**DOI:** 10.1093/jisesa/ieae093

**Published:** 2024-09-30

**Authors:** Clare Boyes, Jennifer K Rowntree, Emma Coulthard

**Affiliations:** Department of Natural Sciences, Manchester Metropolitan University, Manchester, UK; Department of Natural Sciences, Manchester Metropolitan University, Manchester, UK; School of Biological and Marine Sciences, University of Plymouth, Plymouth, UK; Department of Natural Sciences, Manchester Metropolitan University, Manchester, UK

**Keywords:** pollen, agriculture, biodiversity

## Abstract

Declines in pollinating insects have been linked to changes in land cover, affecting the availability of nesting sites and floral resources. Our study is the first analysis of changes in pollen load composition of 2 mining bees, *Andrena barbilabris* (Kirby) and *Andrena flavipes* (Panzer) (Hymenoptera: Andrenidae), at the same sites in central England, over 75 years. This provides a unique opportunity to remove spatial variation and review temporal changes in pollen diet within the context of landscape change. We analyzed modern-day pollen load composition for these species and compared it with historical data from the same sites. We then examined potential links between land-use change and the bees’ diets. Both bees showed dietary flexibility and lower diet breadth for *A. barbilabris*, and the bees’ foraging strategies appear to have changed. *Andrena flavipes* collected more pollen taxa in a single load, while *A. barbilabris* appeared to source pollen from greater distances. Landscape changes at the studied sites have affected the nutritional environment for these bees. Our findings are supported by an existing assessment of floral resources, which found floral diversity has decreased overall in both the habitats used by these bees. However, more research is needed on the nutritional content of pollens used by these bees, both now and historically, to estimate how pollen diversity has changed. The bee’s-eye view underlines the importance of understanding how species respond to local changes so that effective conservation strategies can be developed.

## Graphical Abstract

**Figure:**
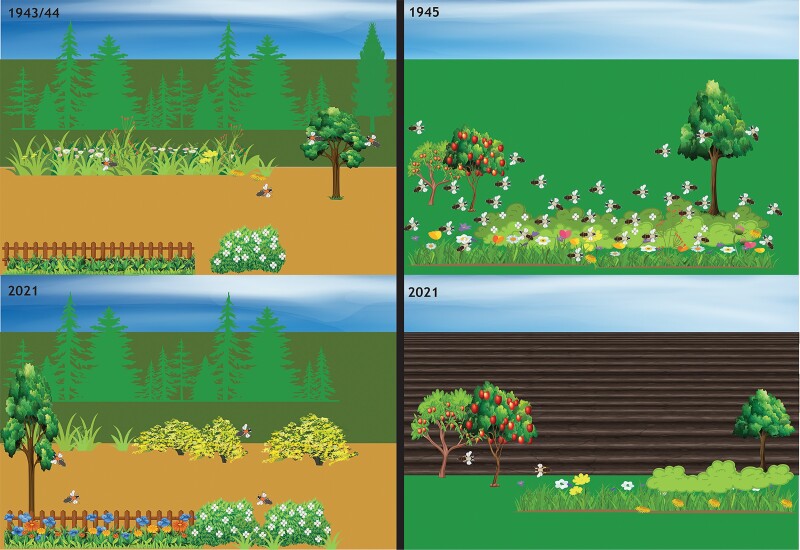
Landscape changes over 80 years at 2 UK sites, appear to be reflected in the pollen diet of 2 *Andrena* bees.

## Introduction

There is mounting evidence that insects are declining globally (Sánchez-Bayo and Wyckhuys 2019, Wagner et al. 2021, Zattara and Aizen 2021) with concern about the impact that this has on ecosystem services such as pollination (Goulson 2019, Cardoso et al. 2020). It has been estimated that 75% of the world’s crops, and almost 90% of flowering plants, rely to some extent on animal pollination (Ollerton et al. 2011). In the United Kingdom, a third of some groups of wild pollinators (bees and hoverflies) have shown declines in their distribution since 1980, with rare species decreasing more than commoner species (Powney et al. 2019). Over the last 30 years, wild bees have continued to decline, with those in areas of high agricultural intensity declining by 4% (Mancini et al. 2023). There are many inter-linked reasons cited for these declines, but the intensification of agriculture, with its associated habitat loss and use of pesticides, is foremost among them (Potts et al. 2010, Goulson et al. 2015, 2018, Raven and Wagner 2021).

Although managed bees are used to pollinate crops, there is evidence that wild bees have an important role to play in the stability of pollination systems, as honeybees alone are not sufficient to maintain agricultural productivity (Garibaldi et al. 2011) and may even have a negative impact on pollination (Page and Williams 2022). Solitary bees are particularly efficient pollinators compared with honeybees (Garibaldi et al. 2013, MacInnis and Forrest 2019), and their contribution to pollinator services has been underestimated (Breeze et al. 2011). Not all wild bees pollinate agricultural crops; however, all bees pollinate wild plants and thus supply some ecosystem services. Not only do pollinators maintain plant diversity (Wei et al. 2012), but they can, for example, improve fruit set, which in turn increases food for wild birds (Jacobs et al. 2009).

Floral resources, including pollen and nectar, are essential for bees’ survival, with nectar providing the energy source to fuel both larval development and adult bees. Pollen is essential for the reproductive success of bees by providing essential nutrients for their developing larvae. In addition, adult bees consume pollen, which has been shown to be essential for egg development in females (Cane et al. 2017). Pollen is highly variable from species to species, both in morphology and nutritional content. The nutritional profiles of different pollens have been found to influence the associated pollinator community, suggesting that bees occupy “nutritional niches” (Vaudo et al. 2024). Research on the nutritional requirements of solitary bees is mainly limited to studies on trap-nesting Megachilidae. However, nutritional ecology provides insights that suggest that a complementary range of pollens is important for the health of generalist bees to satisfy their nutritional requirements, ensuring species fitness (Trinkl et al. 2020, Filipiak et al. 2022).

Bees forage for pollen in different ways. Monolectic bees are specialists which almost exclusively use pollen from one plant species; oligolectic bees collect pollen from a narrow range of species, while polylectic bees are generalists using pollen from at least 4 unrelated families, although they might show a strong preference for one taxon (Müller and Kuhlmann 2008). Diet breadth reflects the number of pollen types used (Cane and Sipes 2006). In bumble bees, species with the greatest declines have the narrowest diet breadth compared with common species (Kleijn and Raemakers 2008). Pollen mixing, where a single load of pollen contains more than one taxon, is more common in solitary bees (Beil et al. 2008, Eckhardt et al. 2014) than honeybees, where individuals tend to exhibit floral constancy on a single foraging trip (Grüter and Ratnieks 2011, Leonhardt and Blüthgen 2012). This might be a way of managing pollens which could be toxic in large quantities (Eckhardt et al. 2014), a response to limited availability of favored pollens (Williams and Tepedino 2003), or a mechanism to provide a balanced diet for successful larval development (Roulston and Cane 2000). The knowledge of pollen preferences in UK solitary bees is mainly taken from the work of Chambers (1968), who collected pollen from ground-nesting bees in England during the 1940s. Chambers’ data has been used to compare historical data with analysis of pollen loads from *Andrena* bees (Hymenoptera: Andrenidae) collected in the contemporary period at various sites throughout central and southern England (Wood and Roberts 2017). This recent study concluded that diet breadth had not changed, but there had been a shift from Rosaceae to Brassicaceae pollen, which was attributed to the removal of hedgerows and the increase in the planting of *Brassica napus* L. (oil-seed rape) since the 2nd World War.

*Andrena* are solitary mining bees and the second most species-rich genus of bees worldwide; they are rapidly speciating (Bossert et al. 2022). This diversity is thought, in part, to be due to their dietary flexibility (Wood 2021). Both our study species nest in loose aggregations and are pollen generalists. *Andrena flavipes* (Panzer) is widely polylectic with 83 different pollen taxa listed on the Database of Pollinator Interactions (DoPI), the highest number of any UK solitary bee (Balfour et al. 2022). *Andrena barbilabris* (Kirby), with 37 taxa is also one of the most widely polylectic UK solitary bees. As generalists they are useful study organisms to explore how changes in the landscape impact their diets. *Andrena flavipes* nests in a variety of soils and as a result is widespread, often occurring in large aggregations. It has 2 generations a year, although only the spring generation was included in this study. It is considered a useful crop pollinator. *Andrena barbilabris* is restricted to sandy soils where it nests in loose aggregations. It flies in early summer and is not believed to be a significant crop pollinator (Else and Edwards 2018). Both bees are thought to have typical foraging ranges of between 100 m and 300 m (Greenleaf et al. 2007).

The floral resources available to bees have changed significantly since the 1940s as agricultural intensification and resulting landscape fragmentation have had significant impacts, with the loss of forage plants cited as a major cause of bee declines (Carvell et al. 2006, Ollerton et al. 2014). An analysis of historic vegetation surveys coupled with an assessment of nectar produced by flowers found arable landscapes were particularly poor for nectar provision, with both low availability and diversity of nectar-producing plants (Baude et al. 2016). A similar analysis has not yet been completed for pollen resources, but Wright et al. (2024) found a significant correlation between pollen and nectar provision both by flower species and at the landscape level (albeit with some exceptions such as *Salix* spp). Therefore, it is reasonable to assume that arable landscapes will be poor for pollen provision.

The aim of our study was to compare the contemporary diet of 2 solitary *Andrena* species with data from the 1940s collected by Chambers (1968) and place this in the context of landscape change. To do this, the original nesting sites were revisited, and pollen was collected from the bees as they returned to their nests. Landscape change was analyzed with historical and contemporary land cover maps using QGIS (QGIS Association 2022). It was expected that the outcome of our study would show similar results to the study of Wood and Roberts (2017), with no change in diet breadth but with similar changes in the relative proportions of different pollen families in the diet linked to changes in farming practice.

## Materials and Methods

### Study Sites

Chambers (1968) studied pollen loads of solitary bees throughout Bedfordshire between 1941 and 1949. Most of these bees were found in a variety of locations in small numbers; however, 2 species were collected in large numbers (>50) from single locations at which they were known to persist to the current day: 130 samples from the spring generation of *A. flavipes* collected in 1945 from Site 1 (Lat: 51°57ʹ23″N, Long: 000°31ʹ40″W); and 83 samples from *A. barbilabris* collected in 1943–1944 from Site 2 (Lat: 51°59ʹ54″N, Long: 000°38ʹ47″W). Site 1 is a narrow road bordered by agricultural land, with some boundary trees and hedging. The bees were nesting in a section of south-facing roadside verge, which covered approximately 90 m^2^. Site 2 was formerly heathland but was planted with coniferous trees for timber in 1778 and has been periodically felled for timber (The Woburn Sands Collection 2019). Here, the bees were nesting in a wide sandy track in an area that covered approximately 100 m^2^. The 2 sites were visited several times between 14-IV-2001 and 16-VI-2021. The timing of visits was constrained due to COVID-19 restrictions in 2020/21.

### Historic Data Collation

Chambers (1968) published summary data of nearly 1,200 pollen loads of *Andrena* bees collected in the 1940s from central England. He did not publish the relative proportions of pollen types in the diet of the bees he studied, but his original notebooks contain this data, and a summary of bee species is included with Wood and Roberts (2017). The pollen analyses from Chambers’ notebooks were digitized by Wood and Roberts and Roberts made a spreadsheet and the original notebooks available for this study.

### Bee Pollen Collection

Site 1 was visited 8 times between 14-IV-2021 and 27-V-2021 with no *A. flavipes* females carrying pollen found on the first or last visit. Site 2 was visited 12 times between 14-IV-2021 and 26-VI-2021 with pollen-laden *A. barbilabris* females found on 5 visits between 8-VI-2021 and 24-VI-2021. To provide a direct comparison with Chambers’ historic work, pollen was collected and analyzed using the methods described by Chambers (1946). Female bees were individually netted as they returned to their nests with pollen. Each bee was placed in a 75 ml plastic flip-top vial with crushed laurel leaves in the bottom for a few seconds until it lost consciousness, after which it was immediately transferred to a clean 2 ml plastic vial to minimize contamination. The vial was kept in a cool, dark bag for up to 90 min. When the bee regained consciousness, it cleaned the pollen from its body and was released. The samples were labeled and kept in a freezer until they were prepared for analysis.

### Botanical Survey

At the start of the field season, a plant survey was carried out within a radius of 500 m of each nesting aggregation. On subsequent visits, all flowering plants were noted. Plants were identified using Stace (2010). A radius of 500 m was chosen as the maximum observed foraging distance reported by Greenleaf et al. (2007). The National Biodiversity Network Atlas (NBN 2022) was used to check distributions for species found in the pollen samples but not recorded during fieldwork. A pollen reference collection of over 150 taxa was created from plants documented by Chambers (1968), and species found flowering during fieldwork, including ornamental varieties where appropriate.

### Pollen Identification

Pollen samples were processed, and slides were prepared, by staining with basic fuchsin and mounting in glycerin jelly. Slides were examined, using light microscopy at ×400, by taking a zigzag course through the slide to review between 500 and 1,000 grains, counting the number of grains of each pollen type (Chambers 1946). Where fewer than 1000 grains were counted, the numbers of grains for all the taxa in each sample were transformed to a proportion of 1,000 grains. As with Chambers’ methods, pollen was identified by consulting the reference collection, and with knowledge of the plants flowering at the time of sample collection. In addition, we consulted Halbritter et al. (2018) and the online pollen library PalDat (Weber and Ulrich 2017). Pollen was grouped into families using Stace (2010). Pollens present at less than 1% of a sample were recorded as a trace in line with Chambers’ (1968) original methods and discounted from further analysis. When pollen could not be identified by species, it was determined by family where possible. Unidentified pollens were present in small quantities in both the historical and contemporary periods and were included in the results as a percentage of total pollen found.

### Land Cover Analysis

Land cover analysis was carried out using QGIS 3.20.3 (2022). The Dudley Stamp land utilization maps are the earliest digital land cover maps for England (Stamp 1948), and ground surveys for the study area were carried out between 1931 and 1935. These maps can be viewed through Digimap (University of Edinburgh 2022). Images of the relevant areas were saved as png files; georeferenced, and digitized. For the current period, the UK Centre for Ecology and Hydrology (UKCEH) 2020 land cover map (Morton et al. 2021) was downloaded through Digimap. The map was compiled from 2020 satellite images. The land cover categories for Dudley Stamp and UKCEH land cover maps are different as the Dudley Stamp map has 15 land cover categories, and the UKCEH map has 21. However, there was insufficient resolution in the historic maps to enable differentiation between some of the Dudley Stamp categories; for example, it was not possible to distinguish between different types of woodland. To assess land cover change, the land cover types were simplified and combined into 6 groups: grassland, woodland, arable, heathland, freshwater, and urban (Supplementary Table S1). The percentage change in the land cover category was calculated within 500 m of the nesting aggregation based on the maximum foraging range of these specific bees (Greenleaf et al. 2007). Additionally, landscape change was calculated at 1, 2, 5, and 10 km radii from the site following work by Senapathi et al. (2015), who chose the smaller radii of 1 and 2 km to cover the average foraging ranges of a suite of pollinators and the distances of 5 and 10 km to provide context for wider landscape change.

### Statistical Methods

All statistical analyses were conducted using R version 4.3.2 (R Core Team 2022). The data for the pollen family does not follow normal distributions and could not be transformed into normality. The data for the pollen family were log-transformed, and Wilcoxon rank-sum was used to test for differences between the time periods (Field et al. 2012). Bonferroni’s correction for multiple comparisons was used to adjust *P*-values to reduce the risk of type-1 errors. A nonmetric multidimensional scaling (NMDS) analysis was performed to visualize differences in pollen usage between the 2 periods, and the difference between the time periods was tested using permutational multivariate analysis of variance (PERMANOVA) in the community ecology package “vegan” version 2.6-4 (Oksanen et al. 2022). Chao species richness of the pollen taxa collected was calculated using vegan’s specpool() function to allow for differences in sample size. Rarefaction and extrapolation curves were plotted in iNEXT version 3.0.1 (Chao et al. 2014, Hsieh et al. 2016); ggplot2 (Wickham 2016) was used to visualize changes in pollen use and NMDS ordinations.

## Results

Chambers analyzed 130 samples of pollen from *A. flavipes* and 83 samples from *A. barbilabris* during the 1940s. In 2021, 30 pollen loads from each species were collected for analysis. It was not possible to collect additional pollen samples due to the small number of bees at both nesting sites. For a full breakdown of pollen use by taxon, both from Chambers’ work and the findings of this study, see Supplementary Table S2.

### Pollen Diet—*A. flavipes*

**Fig. 1.**
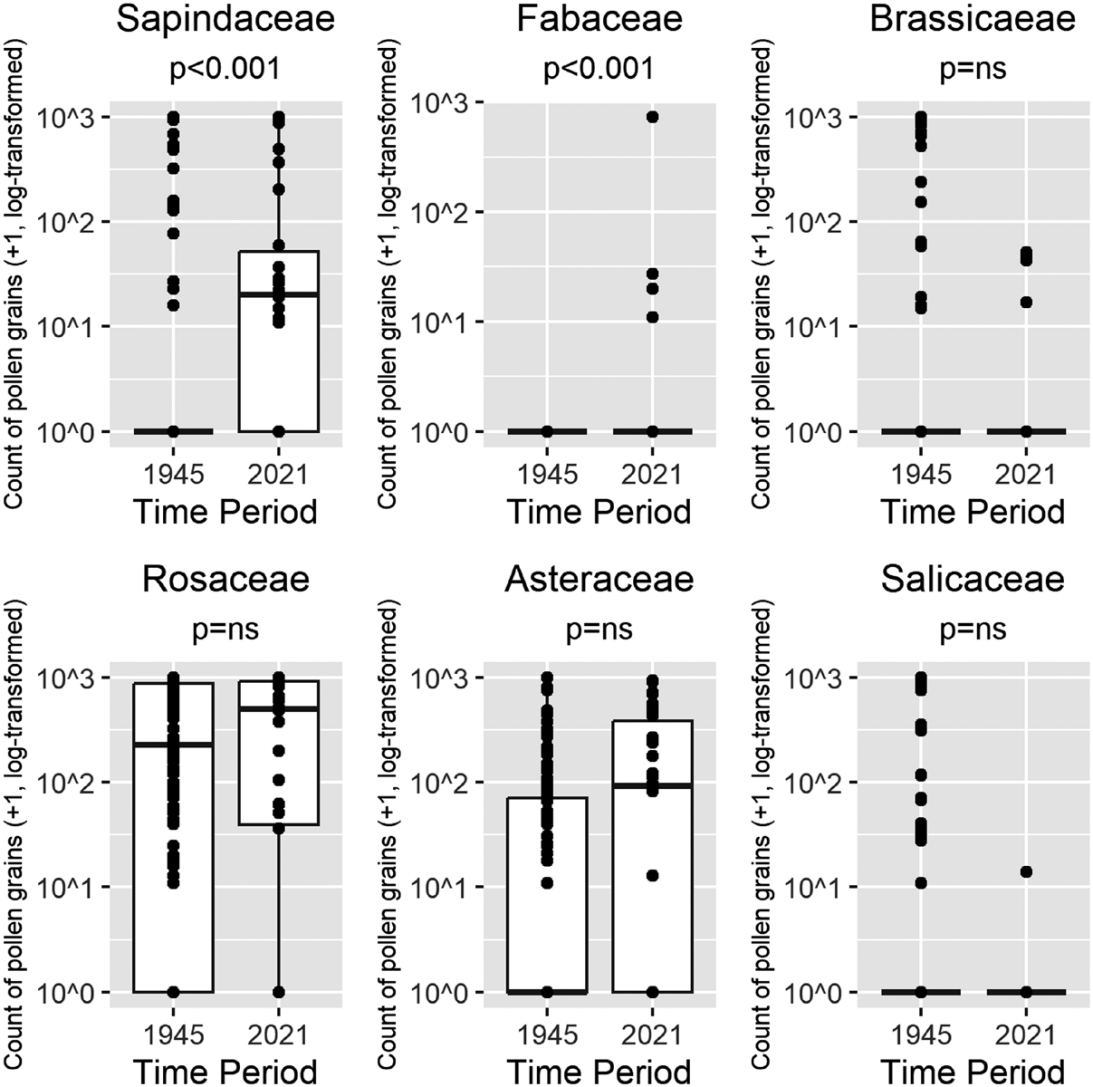
Changes in the use of different pollens for *Andrena flavipes* between 1945 (*n = *130) and 2021 (*n* = 30). Counts are the number of pollen grains + 1 and log-transformed to base 10. The horizontal lines signify the median; the boxes show the interquartile range; the whiskers extend from the box to the minimum and maximum data point if these are within 1.5 times the interquartile range; other values are outliers and are plotted as dots. Significant differences in pollen load composition between time periods were identified using the Wilcoxon rank-sum test with Bonferroni’s correction for multiple comparisons.

Chambers analyzed 130 samples of pollen from *A. flavipes* at Site 1 in April and May 1945. The main source of pollen for *A. flavipes* in the 1940s was Rosaceae with almost 41% of pollen from this family, and this remained an important part of the diet in the contemporary period at 48.2%. A Wilcoxon rank-sum with Bonferroni’s correction demonstrated that proportions of Sapindaceae were significantly higher in the contemporary period (*W* = 1044, *P* < 0.001, effect size *r *= 0.47) (Fig. 1); see Table 1 for the adjusted and unadjusted *P*-values.

**Table 1.** Significant differences in pollen load composition between time periods for *Andrena flavipes* identified using the Wilcoxon rank-sum test with Bonferroni’s correction for multiple comparisons

Family	Original *P*-values		*P*-values with Bonferroni’s correction
Sapindaceae	*W* = 1,044	*P*-value < 0.001	<0.001
Fabaceae	*W* = 1,690	*P*-value < 0.001	<0.001
Amaryllidaceae	*W* = 1,707	*P*-value < 0.001	= 0.006
Betulaceae	*W* = 1,820	*P*-value = 0.003	= 0.049
Asteraceae	*W* = 1,358.5	*P*-value = 0.004	= 0.057
Salicaceae	*W* = 2,348	*P*-value = 0.012	= 0.176
Plantaginaceae	*W* = 2,205	*P*-value = 0.037	= 0.563
Fagaceae	*W* = 1,885	*P*-value = 0.039	= 0.583
Apiaceae	*W* = 1,957.5	*P*-value = 0.926	–
Rosaceae	*W* = 1,767	*P*-value = 0.419	–
Ranunculaceae	*W* = 1,920	*P*-value = 0.877	–
Brassicaceae	*W* = 2,155	*P*-value = 0.201	–
Caryophyllaceae	*W* = 2,073.5	*P*-value = 0.272	–
Euphorbiaceae	*W* = 1,980	*P*-value = 0.503	–
Lamiaceae	*W* = 2,055	*P*-value = 0.197	–

Excluding unknown pollens, 24 pollen taxa were found in the historic pollen samples compared with 16 in 2021. There was some turnover in the pollen taxa found: 14 were found only in the historical period, 10 were common to both periods, and 6 were found only in the contemporary period. For a breakdown of the pollen taxa found in Chambers’ work and this study, see Supplementary Table S3. A Wilcoxon rank-sum test demonstrates that the average species richness per pollen load was significantly higher in the contemporary (3.3) compared to the historic (2.4) period (*W* = 1337.5, *P* = 0.006), while the proportion of single-pollen loads was significantly lower (13% compared to a historic 31.5%).

Chambers recorded the time he spent collecting pollen samples from *A. flavipes* at Site 1 in his notebooks. A Wilcoxon rank-sum test showed a significant reduction in the number of samples taken. Chambers averaged 36 *A. flavipes* samples per hour, compared with an average of 2 samples per hour in 2021 (*W* = 30, *P* = 0.007).

### Pollen Diet—*A. barbilabris*

**Fig. 2.**
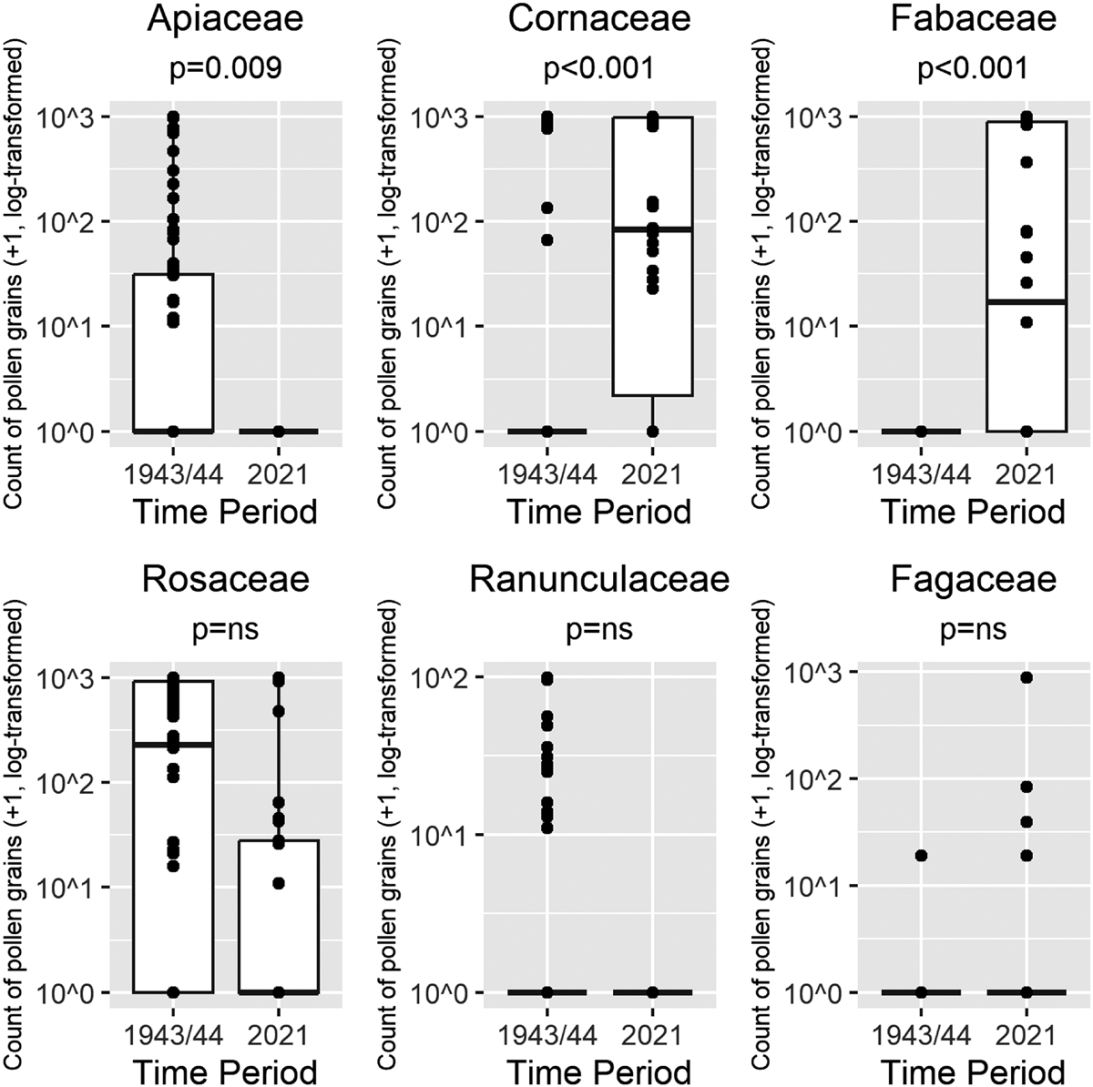
Changes in the use of different pollens for *Andrena barbilabris* between 1943 and 1944 (*n* = 83) and 2021 (*n* = 30). Counts are the number of pollen grains + 1 and log-transformed to base 10. The horizontal lines signify the median; the boxes show the interquartile range; the whiskers extend from the box to the minimum and maximum data point if these are within 1.5 times the interquartile range; other values are outliers and are plotted as dots. Significant differences in pollen load composition between time periods were identified using the Wilcoxon rank-sum test with Bonferroni’s correction for multiple comparisons.

The diet of *A. barbilabris* differed between the 2 periods, and a Wilcoxon rank-sum test with Bonferroni’s correction demonstrated that the use of Apiaceae pollen was significantly less in the contemporary period (*W* = 1635, *P* = 0.009, effect size *r* = 0.31) (Fig. 2). In contrast, the use of Cornaceae was significantly more (*W* = 598, *P *< 0.001, effect size *r* = 0.45) (Fig. 2), and this was the most prevalent pollen in 2021, being present in 22/30 loads and accounting for 40.7% of all pollen found. The use of Fabaceae pollen was also significantly more in the contemporary period (*W* = 581, *P* < 0.001, effect size *r* = 0.64) (Fig. 2), being present in 10/30 loads and comprising 33.6% of the pollen identified. See Table 2 for the adjusted and unadjusted *p*-values. Rosaceae pollen made up 11.8% of the pollen collected, and along with Cornaceae and Fabaceae comprised 86% of the pollen taxa found in 2021.

**Table 2.** Significant differences in pollen load composition between time periods for *Andrena barbilabris* identified using the Wilcoxon rank-sum test with Bonferroni’s correction for multiple comparisons

Family	Original *P*-values		*P*-values with Bonferroni’s correction
Cornaceae	W = 598	*P*-value < 0.001	<0.001
Apiaceae	W = 1,635	*P*-value < 0.001	= 0.009
Fabaceae	W = 581	*P*-value < 0.001	<0.001
Rosaceae	W = 1,666	*P*-value = 0.003	= 0.054
Sapindaceae	W = 1,497	*P*-value = 0.018	= 0.296
Ranunculaceae	W = 1,485	*P*-value = 0.010	= 0.163
Fagaceae	W = 1,092.5	*P*-value = 0.006	= 0.089
Brassicaceae	W = 1,334	*P*-value = 0.405	–
Asteraceae	W = 1,365	*P*-value = 0.081	–
Pinaceae	W = 1,218	*P*-value = 0.451	–
Salicaceae	W = 1,290	*P*-value = 0.299	–
Polygonaceae	W = 1,260	*P*-value = 0.561	–
Aquifoliaceae	W = 1,219	*P*-value = 0.468	–
Caryophyllaceae	W = 1,275	*P*-value = 0.401	–

Twenty-eight pollen taxa were identified in the historic period compared with 12 in 2021. There was turnover in the pollen taxa found in the samples: 21 taxa were found only in the historical period, 7 were common to both periods, and 5 were found only in the contemporary period. The average species richness of each *A. barbilabris* pollen load remained at 2, and the proportion of single-pollen loads remained constant at around 37%. For a breakdown of the pollen taxa found in Chambers’ work and this study, see Supplementary Table S4.

**Fig. 3.**
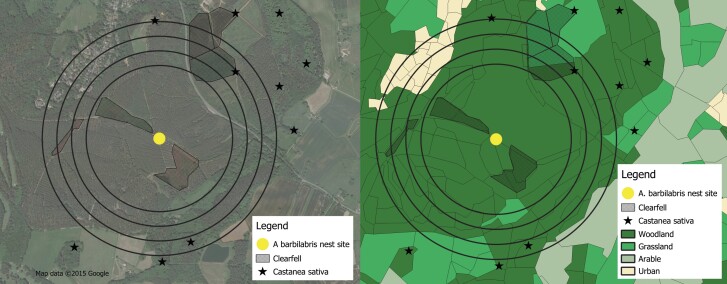
A) Satellite imagery (Google Maps) and B) Land cover classes (based on CEH 2021 land cover maps) of *Andrena barbilabris* nest site on Site 2, with buffers shown at 500 m, 600 m, 700 m, and 800 m. Areas of recent clear-fell and *Castanea sativa* trees were marked. Maps produced using QGIS 3.20.3 (2022).

Many of the pollen taxa used by *A. barbilabris* in 2021 were not found near the nesting aggregation during the botanical surveys. For example, the closest *Castanea sativa* Mill. (sweet chestnut) was growing almost 700 m from the nest site (NBN 2022). In addition, it was estimated from satellite imagery using QGIS that 9.6% of the land within 500 m of the nest site had recently been clear-felled. Work on the westernmost area of the site was taking place in 2021 whilst the fieldwork for the study was being carried out (Fig. 3A and B).

### Diet Breadth

**Fig. 4.**
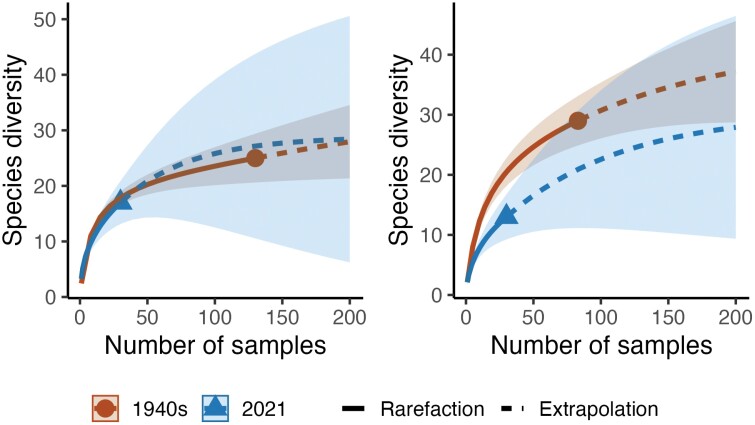
A) Species accumulation curves of the pollen genera collected by *Andrena flavipes* between 1945 (*n* = 130) and 2021 (*n* = 30). B) Species accumulation curves of the pollen genera collected by *Andrena barbilabris* between 1943 and 1944 (*n* = 83) and 2021 (*n* = 30)**.**

Rarefaction and extrapolation curves of the pollen genera collected by the bees were plotted in iNEXT (Fig. 4A and B). *Andrena. barbilabris* had a narrower diet in 2021, with a Chao species richness of 42 ± 12 (± SE) in the 1940s compared to 30 ± 22 in 2021. Although Chao species richness for *A. flavipes* was 43 ± 23 in the 1940s compared with 29 ± 13 in 2021, the species accumulation curve does not demonstrate a narrower diet breadth.

**Fig. 5.**
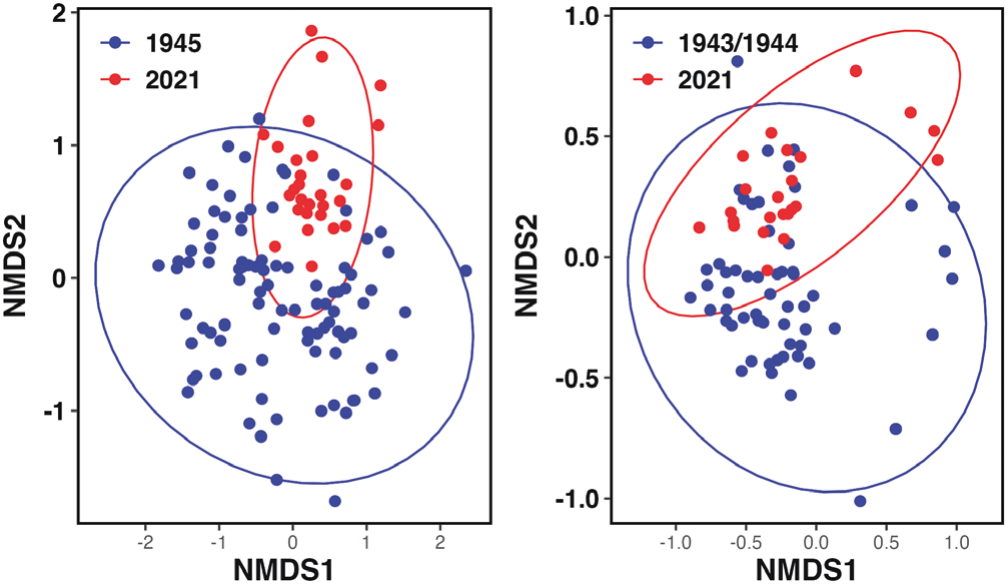
A) Differences in pollen load composition of *Andrena flavipes* between 1945 (*n* = 130) and 2021 (*n* = 30). Analysis of Permutational multivariate analysis of variance (PERMANOVA) results: *F*_1_ = 11.29, *P* = 0.001. B) Differences in pollen load composition of *Andrena barbilabris* between 1943/1944 (*n* = 83) and 2021 (*n* = 30). Permutational multivariate analysis of variance (PERMANOVA) results: *F*_1_* *= 10.14, *P* = 0.001. Each point represents a single-pollen load from one bee, and these are color coded according to the sampling period. Ellipses show 95% CI.

Differences in pollen load composition between the time periods were visualized using nonmetric multidimensional scaling (NMDS) (Fig. 5A and B). The plots overlap, indicating similarity in the host plant use. However, there is a wider spread of points in the earlier period, perhaps suggesting a wider diet breadth. PERMANOVA was highly significant (*P* = 0.001) for both species, as is beta dispersion (*A. flavipes: F*_*1*_ = 25.14, *P* = 0.001; *A. barbilabris: F*_*1*_ = 18.18, *P* = 0.001). Chi-squared test of association revealed that woody plants (trees and shrubs) made up a significantly higher proportion of the pollen diet of both bees changing from 61.7% to 65.4% (𝜒^2^_*1*_ = 143.32, *P* < 0.001) in *A. flavipes* and from 76.6% to 89.7% (𝜒^2^_*1*_ = 2355, *P* < 0.001) in *A. barbilabris*.

### Land-Use Change

Landscape mapping demonstrates that the landscapes around the sites have changed substantially since the 1940s, particularly the reduction of grassland by conversion to arable land. At Site 1, grassland changed from 90.9% to 16.1% within a 500 m radius of the nesting site. Conversely, arable land expanded from 3.5% to 78.2% land cover at a 500 m radius. At Site 2, the changes were less marked. There was little change at 500 m; at 1 km from the nest site the main changes were a small reduction in woodland and a small increase in grassland. Increasing urbanization was not a major factor within 1 km of either bee aggregation, although it was a factor at the wider landscape scale of 10 km. See Tables 3 and 4 for further details.

**Table 3.** Percentage land cover (diameter from a central point) at Site 1, 1930s Dudley Stamp maps and UKCEH 2020 land cover map (m = meters; km = kilometers)

	Percentage 1930s land use					Percentage 2020 land use				
	**500 m**	**1 km**	**2 km**	**5 km**	**10 km**	**500 m**	**1 km**	**2 km**	**5 km**	**10 km**
Woodland	1.9	10.7	6.0	7.9	9.7	2.3	2.4	3.3	7.6	10.2
Arable	3.5	20.1	22.9	27.0	30.3	78.2	60.6	60.9	56.8	44.5
Grassland	90.9	54.7	62.0	57.7	46.9	16.1	21.2	23.8	27.6	26.4
Heathland			0.3	0.9	1.9					
Urban	3.7	14.5	8.8	6.5	11.2	3.4	15.6	11.7	7.8	18.6
Other							0.2	0.3	0.2	0.3

**Table 4.** Percentage land cover (diameter from a central point) at Site 2. 1930s Dudley Stamp maps and UKCEH 2020 land cover map (m = meters; km = kilometers)

	Percentage 1930s land use					Percentage 2020 land use				
	**500 m**	**1 km**	**2 km**	**5 km**	**10 km**	**500 m**	**1 km**	**2 km**	**5 km**	**10 km**
Woodland	100	79.8	48.9	25.0	10.3	98	74.2	41.8	20.3	11.3
Arable		5	11.9	12.3	17.3	0.8	5.7	20.2	29.9	36.8
Grassland		7.2	21.4	51.5	62.3	1.2	14.3	24.9	32.2	28.7
Heathland			4.5	1.9	1.1					
Urban		8.0	13.3	9.3	9.0		5.8	12.4	16.5	22.0
Other								0.7	1.1	1.2

## Discussion

Despite growing evidence of the importance of solitary bees in providing pollination services, few studies have investigated how wild bees respond to landscape changes. There are records of mining bee aggregations persisting at sites for up to 6 decades (Danforth et al. 2019). However, the bees studied here are noteworthy in that the species were nesting at these sites almost 80 years ago, and there are historic records of the pollen load composition from that time. Our analysis demonstrates that both bees exhibited dietary flexibility, taking a wide range of pollens from different families, but pollen load composition differed over time. Our expectation was that the diet breadth of our study bees would be broadly similar between the 2 time periods, but *A. barbilabris* appeared to have a narrower diet in 2021.

Access to floral resources is the most important limiting factor for wild bees, and dietary preferences will affect their populations (Roulston and Goodell 2011). It has been shown that a high turnover in plant-pollinator interactions over time is not uncommon (Jacquemin et al. 2020) and that bees that exhibit dietary flexibility are better equipped to respond to landscape changes (Lami et al. 2021). In addition, polylectic bees appear to obtain fitness benefits from their varied pollen diet (Woodard and Jha 2017). Although it is difficult to generalize with only 2-time points, both bees in this study demonstrated dietary flexibility, using sources of pollen not used historically.

As predicted, landscape change affected the diet of both bees, but this appeared to be operating at different scales. *Andrena flavipes* occurred at a site that had seen a significant change from grassland to intensive arable farmland. Here, the modern diet reflected the loss of pollen from hedgerow shrubs, probably due to poor hedgerow conditions. *Andrena barbilabris* showed more pronounced dietary changes, yet the landscape changes at Site 2 were minimal. However, pollen analysis coupled with the botanical surveys suggests a loss of ground flora at the site, and a third of the pollen found was from a novel resource. Our results also suggest that the foraging strategies of both bees changed: in the contemporary period, *A. flavipes* collected more types of pollen in a single load, and *A. barbilabris* appeared to source pollen from greater distances. The finding that, in 2021, *A. barbilabris* was obtaining 65% of its pollen from sources growing more than 500 m from its nests was unexpected. Our findings highlight that landscape mapping only gives part of the picture: finer detail is needed to understand the nuances of how changes impact individual species.

Bee species operate within a “nutritional niche” whereby landscape floral resources provide an optimal pollen diet (Parreño et al. 2022). This niche consists of a precise mixture of nutrients enabling peak species fitness. Within this niche, there are crucial nutrients without which the bees cannot survive; however, generalist species can tolerate a suboptimal diet if some less critical nutrients are deficient in their environment, resulting in a wider nutritional niche than more specialist species. Pollen may be rich in proteins but lack essential amino acids or have an otherwise unbalanced nutritional profile, suggesting that a single-pollen taxon might not meet all of a bee’s nutritional needs (Chau and Rehan 2024). Theoretically, a very species-rich habitat could be nutritionally poor if all the pollens have a similar chemical profile, resulting in a homogenous nutritional environment. To complicate matters, phylogenetically related plants do not always have similar nutritional profiles, although sterol content does seem to be more closely aligned to plant phylogeny than other nutrients (Zu et al. 2021). Landscapes, therefore, need a range of pollens with different nutritional profiles, providing complementary floral resources to support a diverse bee community, i.e., pollen rather than species diversity (Vaudo et al. 2024).

### Use of Toxic Pollen

Despite their broad polylecty, the species differed in the pollens they collected. *Andrena flavipes* used Asteraceae pollen, particularly *Taraxacum*, which constituted 21.6% of the pollen collected in 2021. Many generalist bees avoid Asteraceae pollens, whilst other related bees specialize on this taxon, which has been termed the “Asteraceae Paradox” (Müller and Kuhlmann 2008). A number of studies have shown the negative effects of *Taraxacum* pollen on larval development in both solitary and social bees (Tasei and Aupinel 2008). Whilst the exact mechanism for this remains unclear, *Taraxacum* pollen is rich in protein but deficient in some essential amino acids (Roulston and Cane 2000), and may also contain a chemical deterrent such as uncommon sterols (Zu et al. 2021) or alkaloids (Vanderplanck et al. 2020a). Along with the higher use of toxic pollen, the average number of taxa per pollen load was greater in the recent period. Pollen mixing is thought to mitigate the impact of toxic pollen on larval development (Eckhardt et al. 2014). In contrast, there were only trace amounts of *Taraxacum* pollen in the *Andrena barbilabris* samples from both periods, even though *Taraxacum* was growing close to the nest aggregation in 2021, suggesting that *A. barbilabris* avoids this pollen.

### Andrena flavipes

In both periods, the most-used family of pollens by *A. flavipes* was Rosaceae, probably linked to a small orchard in the garden next to the nest site. There were small changes in Rosaceae pollen (up 7.5 percentage points to 48%) and Brassicaceae (down 13 percentage points to 0.5%), contrasting with the trends reported by Wood and Roberts (2017). Baude et al. (2016) reported that linear features such as hedges and road verges were important for higher levels of floral resources, but at Site 1, many of the surrounding hedges were in poor condition due to mechanized cutting. Hedgerows in arable systems are between 20% and 30% less dense than in pastoral systems (Robinson and Sutherland 2002) and, at this site, resulted in some hedgerow plants failing to flower in the year of the survey.

### Foraging Distances

A number of studies have assessed the foraging distance of solitary bees, with body length giving an indication of foraging distances (Gathmann and Tscharntke 2002). Further research by Greenleaf et al. (2007) found that the average intertegular span, i.e., the distance between the wing bases, was a more useful predictor of foraging distance than body length. The intertegular span for *A. flavipes* is 2.2 mm, which predicts a typical foraging range of 300 m and an observed maximum of 500 m. All the pollen taxa found in the *A. flavipes* 2021 samples were found during the botanical survey growing within 100 m of the nests, well within its predicted foraging range. The slightly smaller *A. barbilabris* has an average intertegular span of 2.1 mm which also predicts a typical range of 300 m and a maximum observed range of 500 m (Greenleaf et al. 2007). Using translocation experiments, Gathmann and Tscharntke (2002) estimated that only 10% of *A. barbilabris* females returned to their nests from a distance of 530 m. Yet in our study, 27 of 30 pollen loads contained a proportion of pollen, which appeared to have originated at least 575 m from the nest site. For example, *Cornus* spp (dogwood) was not found during the botanical surveys but was most likely growing in gardens at least 600 m from the nest site. Similarly, *Castanea sativa*, *Prunus* spp. and *Brassica* spp. which were found in the contemporary pollen samples, were absent from the botanical surveys. These plants were all growing more than 575 m from the nest site with, for example, *Brassica* spp. and *Prunus* spp. observed in the vegetable gardens of distant houses. Traces of pollen from ornamental plants were found in some samples, e.g., *Lilium* and *Knautia*, which suggests that *A. barbilabris* was foraging in gardens.

It is impossible to be definitive about the distances *A. barbilabris* was flying to collect pollen in the 1940s, although approximately 80% of the pollen taxa noted historically are from plants found during the 2021 botanical survey within 500 m of the nesting aggregation. By carrying out pollen analysis and mapping identified pollens to plants in the landscape, bees had longer foraging ranges than predicted by body size alone (Beil et al. 2008). However, Zurbuchen et al. (2010b) argued that maximum foraging distances were much less important for understanding bee foraging dynamics than the average foraging distance, as only a few bees in each population will be able to successfully forage at greater distances. As a high proportion of the sampled *A. barbilabris* were foraging at long distances, this suggests that the pollen diversity in the area close to the nesting site did not meet the bees’ nutritional requirements. This is likely to have a fitness cost with impacts such as reduced immune response, fewer offspring, sex imbalances with more males produced, and increased brood parasitism (Peterson et al. 2006, Zurbuchen et al. 2010a, Seidelmann 2014). The botanical survey on Site 2 found few flowering herbaceous plants when the bees were active. Plants in the Apiaceae, which made up 9.5% of the pollen diet in the historical period, had completely disappeared from the pollen diet and were not found on the botanical surveys. The loss of ground flora may be contributing to the long foraging distances found in this study and could impact the survival of this species at this site.

### *Andrena barbilabris*—Novel Pollens

The *A. barbilabris* samples contained 2 novel pollens, both of which have been used to test the nutritional content of bumble bee diets. *Cytisus scoparius* L. (broom) formed a third of the pollen collected by bees in the contemporary samples but was not present in the historic samples. It appears to be uncommonly used by solitary bees (Balfour et al. 2022). *Cystisus scoparius* was abundant on Aspley Heath, growing within a few meters of the nests, so it is perhaps surprising that this did not form a larger proportion of the collected pollen, especially considering the bees’ long foraging distances. There are a few possible explanations. *C. scoparius* pollen is high in protein (de Sá-Otero et al. 2009) and, when used in a bumble bee feeding experiment resulted in the heaviest larvae of all the experimental diets. However, there was some adult bee mortality, which could be related to alkaloids present in the pollen (Moerman et al. 2017) or due to a high percentage of free amino acids (Vanderplanck et al. 2014). *Cytisus* pollen is also low in total sterol content and has a different sterolic profile compared with commoner pollens such as those from the Rosaceae family (Vanderplanck et al. 2014, 2020c). The types and levels of sterols in pollen are important for bees, which require these compounds for several metabolic processes. As they cannot manufacture sterols themselves, they must be obtained from plants. *Cytisus scoparius* pollen may not be well metabolized by many polylectic bees (Vanderplanck et al. 2020b), so it could be an unsuitable pollen for this species. However, another possibility is that the bees can metabolize these sterols and were using *C. scoparius* instead of *Acer pseudoplatanus* L. (Sycamore), which was historically present in the pollen samples and has a similar sterol profile to *Cytisus* (Vanderplanck et al. 2020c).

Alternatively, *C. scoparius* pollen may be compromised by organisms which could be harmful to the developing bee larvae. Flowers can also act as a source of disease for bees with spread of pathogens between bee species (Graystock et al. 2013, 2015, Ravoet et al. 2014). The samples with a high proportion of *C. scoparius* pollen appeared to contain large numbers of microorganisms (such as fungal spores and bacteria), which could potentially exploit the pollen stores in the nests. Perhaps the bees were avoiding *C. scoparius* pollen because it was harmful to the larvae in some way.

The second novel pollen used in 2021 by *A. barbilabris* was *Castanea sativa* pollen. This had been thought to be wind-pollinated but has recently been confirmed as entomophilous (Petit and Larue 2022). In a bumble bee feeding experiment, this small-grained pollen performed very well with a high pollen efficacy and low larval and worker mortality (Tasei and Aupinel 2008). The reward for foraging this high-quality pollen, therefore, could outweigh the risks of longer foraging distances for those bees able to forage at this distance.

The use of *Cornus* spp. pollen by *A. barbilabris* constituted 40.7% in 2021. This is a large-grained pollen, so our methods will have underestimated the value of *A. barbilabris*. *Cornus* seems to be uncommonly used by solitary bees, with only a few species listed on the DoPI (Balfour et al. 2022). It was not used by *A. flavipes*, even though it was flowering at site 1 during fieldwork. As a widespread shrub with open flowers, this suggests that *Cornus* might have some biochemical properties that make it unsuitable for many bees, and it also hints that the nutritional niches of these 2 widely polylectic bees are not identical. As far as we can tell, the nutritional content of this pollen is unknown, so we are unable to draw any firm conclusions.

More research is certainly needed on the nutritional content of a wider range of common pollens used by solitary bees and their dietary requirements. However, the nutritional diversity of the landscape was almost certainly closer to optimal at the time of Chambers’ work, as this was before postwar agricultural intensification, and the bees still survive at these sites (albeit in reduced numbers). Chambers’ work, therefore, provides an invaluable resource about the dietary requirements of Andrena bees, and ensuring that pollen diversity reflects that found historically would be helpful. His methods offer a practical way to assess bees’ nutritional niches by collecting pollen directly from the bees.

### Landscape change

Habitat fragmentation is a significant cause of pollinator decline as changes in land cover significantly affect pollinator communities. (Senapathi et al. 2015). The landscape around the sites has changed significantly since the 1940s, with an increase in arable land linked to postwar changes in agriculture and increasing urbanization. However, gardens are important for bumble bee survival (Goulson et al. 2010), and established gardens at both sites were inferred to be important for both species.

The change in diet breadth most likely reflects local changes in the landscape since the 1940s. Baude et al. (2016) investigated the changing trends in floral resources in Britain which included measures of nectar diversity from 1978. Nectar diversity can be taken as a proxy for diet breadth. Of particular relevance are their findings that neutral grassland is one of the best habitats for nectar diversity, whereas arable land is poorest, and that both arable land and coniferous woodland (Site 2) have shown declines in nectar diversity since the 1970s. Their work supports our findings by demonstrating that the breadth of floral resources has declined since the 1930s in the habitats relevant to the bees in our study.

In his personal notebooks, Chambers recorded the time spent collecting *A. flavipes* samples at Site 1. On one occasion, Chambers was able to collect 28 pollen samples in 30 minutes. However, he reported that the decline of rabbits following myxomatosis resulted in rank vegetation on the site, which made it less suitable. Numbers had already declined by 1949, and now the nests are restricted to the bottom 30 cm of the bank, which is relatively clear of vegetation. This suggests that the nesting site is a limiting factor for the population of *A. flavipes* at site 1 although it also is possible that there are dietary restraints as well.

Comparing the landcover maps for the 2 periods, Site 2 has remained largely wooded. However, it is likely there have been changes to the woodland structure due to regular cycles of planting and clear-felling. With only small landscape changes, it was unexpected that the diet of this bee had changed significantly compared with that in the 1940s. Baude et al. (2016) suggested that increased growth of conifers could shade out ground flora, leading to a reduction in nectar diversity, but at Site 2, the main track in which the bees were nesting was wide and not heavily shaded. However, the site is used extensively for recreational purposes, as well as by forestry machinery used for clear-felling, and this may impact the bees’ success by disturbing the nests. This heavy footfall, possibly exacerbated by intensive use during the Covid-19 pandemic, could be a possible explanation for the disappearance of many herbaceous plants from the site. The recent clear-fell within 150 m of the nesting site (Fig. 3A and B) could also explain why the bees were foraging further afield if plants that had previously been used were no longer present in the vicinity of the nests. Here, the bee’s-eye view highlights local changes that are not apparent at the landscape scale and highlights the importance of understanding pollen diversity and how this impacts solitary bees’ responses to local changes. The fact that these species persist at sites where they have been recorded for almost 80 years, even though the floral resource appears suboptimal, suggests that the importance of familiar nesting sites is underestimated, and further research is needed on this topic.

UK agri-environment schemes have encouraged the planting of wildflower strips to provide floral resources for pollinators, although these have had limited success (Wood et al. 2015, 2017, Nichols et al. 2022). They generally provide floral resources in July and August, after the main flight period of the spring-flying *Andrena* species studied here. However, they have been shown to be important for summer-flying solitary bees in apple orchards (von Königslöw et al. 2022). Nichols et al. (2019) investigated the best wildflower species for solitary bees and created a list of the 14 top-ranking wildflowers. From this, only *Taraxacum* was growing at either site, reflecting the paucity of floral resources available.

### Use of Pollens from Woody Plants

In the absence of forbs, both species were using pollen from woody plants. In 2021, almost 90% of the pollen collected by *A. barbilabris* and 65% collected by *A. flavipes* was from woody plants, which were significantly higher proportions than the historic period. Less than 1% of the pollens were from wind-pollinated trees, so anemophilous pollens did not appear to be significant for these bees. This contrasts with some species that are dependent on oak pollen, e.g., *Osmia bicornis* L. (Persson et al. 2018, Eckerter et al. 2022). Tree pollen is increasingly recognized as important for solitary bees as it provides a concentrated resource (Donkersley 2019, Allen and Davies 2023) and has been shown to provide essential nutrients for larval development (Filipiak 2024). In Chambers’ original data, the proportion of pollen from woody plants taken by all species was almost half, with over 30% of the total pollens from woody Rosaceae (see Supplementary Table S5). The importance of trees for pollinators has been underestimated and is overlooked in many of the agri-environment schemes to support pollinators (Alison et al. 2022). The English Environmental Land Management Scheme, with its focus on local nature recovery and landscape recovery, aims to improve habitat diversity. It, therefore, provides an opportunity to enhance floral resources by selecting trees and shrubs for planting schemes which would benefit pollinators, particularly in spring and early summer (e.g., Rosaceae and Sapindaceae) and improving hedgerow management to allow adequate flowering.

### Experimental Considerations

Analysis of pollen diet has become more sophisticated since Chambers (1968) carried out his work. Correction for pollen grain size is considered important for calculating the relative importance of different pollens in the diet (Buchmann and O’Rourke 1991, da Silveira 1991). This method was used by Wood and Roberts (2017), who also made an adjustment to their calculations by estimating the relative size of the pollen load, i.e., whether there appeared to be a full load or a portion of a load. However, these techniques were not used by Chambers. Therefore, some caution should be used when comparing our findings with those of other workers, as their methods may not provide a direct comparison with the proportions of different pollens found in the diet between the historical and contemporary periods.

The historic maps did not provide sufficient resolution to distinguish between different woodland types, so it was not possible to determine whether the composition of the woodland at Site 2, i.e., the proportion of coniferous and deciduous trees, had changed between the 2 time periods. This was disappointing as our study demonstrated that pollen from deciduous trees is important for the bees studied.

Covid-19 restrictions in place during 2020–2021 delayed fieldwork from mid-March 2020 until mid-April 2021, so it is possible that some of the early flight period of both bees was missed. However, the cool, wet spring (Met Office 2021) resulted in pollen-laden bees not being found on the early visits to the sites, suggesting that the flight period was delayed. This may have resulted in some flowers having finished by the time females were on the wing, e.g., *Salix* at Site 1 and *Crataegus* at Site 2. Both samples were smaller than planned for 3 reasons: firstly, the difficulty of timing fieldwork to periods of suitable weather; secondly, Covid-19 restrictions; and thirdly, the small number of bees at the nesting sites.

The restriction of this case study to 2 species and sites results in reduced generalizability. However, the opportunity to sample additional sites was limited by Chambers’ original data, which mainly contained data for very small numbers of bees at each site across the area in which he was working. Further study of these 2 species at these sites in future years would help to improve our understanding of how these bees are adapting to a changing nutritional landscape.

## Supplementary Material

ieae093_suppl_Supplementary_Table_S1

ieae093_suppl_Supplementary_Table_S2

ieae093_suppl_Supplementary_Table_S3

ieae093_suppl_Supplementary_Table_S4

ieae093_suppl_Supplementary_Table_S5

## Data Availability

Data are archived at Figshare: 10.6084/m9.figshare.26031202
