# Supplementary material for: A bee’s-eye view of landscape change: differences in diet of 2 Andrena species (Hymenoptera: Andrenidae) between 1943 and 2021
Source: J Insect Sci. 2024 Sep 30;24(4):27. doi: 10.1093/jisesa/ieae093 (PMC11441578; doi:10.1093/jisesa/ieae093)
Supplement: ieae093_suppl_Supplementary_Table_S1 [file ieae093_suppl_supplementary_table_s1.docx]

Supp. Table S1

Land Cover Categories showing reclassification of UKCEH (2020) land cover class, and Dudley Stamp land cover categories (after Senapathi, 2015)

| Land cover identifier | UKCEH land cover class (2020) | Dudley Stamp land cover category (1931-1935) | Reclassified category |
| --- | --- | --- | --- |
| 1 | Deciduous woodland | Forest and woodland | Woodland |
| 2 | Coniferous woodland |  |  |
| 3 | Arable | Arable land | Arable |
| 4 | Improved grassland | Meadowland and permanent grass | Grassland |
| 5 | Neutral grassland |  |  |
| 6 | Calcareous grassland |  |  |
| 7 | Acid grassland |  |  |
| 8 | Fen* | Heath and moorland | Heathland |
| 9 | Heather |  |  |
| 10 | Heather grassland |  |  |
| 11 | Bog* |  |  |
| 12 | Inland rock* |  |  |
| 13 | Saltwater* | Water | Freshwater |
| 14 | Freshwater |  |  |
| 15 | Supralittoral rock* | Coastal* | Not relevant |
| 16 | Supralittoral sediment* |  |  |
| 17 | Littoral rock* |  |  |
| 18 | Littoral sediment* |  |  |
| 19 | Saltmarsh* |  |  |
| 20 | Urban | Gardens etc  Land agriculturally unproductive | Urban |
| 21 | Suburban |  |  |

*Habitat not present within current study area
