# Supplementary material for: A bee’s-eye view of landscape change: differences in diet of 2 Andrena species (Hymenoptera: Andrenidae) between 1943 and 2021
Source: J Insect Sci. 2024 Sep 30;24(4):27. doi: 10.1093/jisesa/ieae093 (PMC11441578; doi:10.1093/jisesa/ieae093)
Supplement: ieae093_suppl_Supplementary_Table_S2 [file ieae093_suppl_supplementary_table_s2.docx]

Supp. Table S2

# Host plant use for *Andrena barbilabris* and *Andrena flavipes* in the historic and contemporary period.

Plant family: AMA, Amaryllidaceae; API, Apiaceae; AST, Asteraceae; BRA, Brassicaceae; COR, Cornaceae; PLA, Plantagineae;

RAN, Ranunculaceae, ROS, Rosaceae; SAL, Salicaceae; SAP, Sapindaceae; u/k, unknown.

| Species | Number of  pollen loads | Time period | Proportion of pollen grains by family (%) | % pure loads of  most-used pollen | % loads containing most-used host |
| --- | --- | --- | --- | --- | --- |
| *Andrena barbilabris* | 83 | 1943-1944 | ROS 42.2, SAP 16.1, COR 14.9, BRA 11.3, API 9.5, SAL 1.6,  others 2.0, u/k 2.3 | 18.1 | 60.2 |
| *Andrena barbilabris* | 30 | 2021 | COR 40.7, FAB 33.6, ROS 11.8, BRA 10.0, FAG 3.2,  others 0.3, u/k 0.4 | 16.7 | 73.3 |
|  |  |  |  |  |  |
| *Andrena flavipes* | 130 | 1945 | ROS 40.7, BRA 13.6, SAL 12.5, RAN 10.8, SAP 8.2, AST 7.8,  PLA 4.4, others 1.9, u/k 0.1 | 13.8 | 70.8 |
| *Andrena flavipes* | 30 | 2021 | ROS 48.2, AST 21.7, SAP 17.3, RAN 3.2, API 3.0, FAB 2.6, AMA 2.4,  others 1.5, u/k 0.1 | 6.7 | 76.7 |
