# Supplementary material for: A bee’s-eye view of landscape change: differences in diet of 2 Andrena species (Hymenoptera: Andrenidae) between 1943 and 2021
Source: J Insect Sci. 2024 Sep 30;24(4):27. doi: 10.1093/jisesa/ieae093 (PMC11441578; doi:10.1093/jisesa/ieae093)
Supplement: ieae093_suppl_Supplementary_Table_S3 [file ieae093_suppl_supplementary_table_s3.docx]

Supp. Table S3

## Pollens in diet of *Andrena flavipes* and flowering plants found historically and during botanical survey at Site 1 April-May 2021

| *Family* | *Species* | Common name | Total proportion of pollen in 1945 samples | Total proportion of pollen in 2021 samples | Observed during 2021 botanical survey |
| --- | --- | --- | --- | --- | --- |
| Rosaceae | *Crataegus monogyna* | Hawthorn | 20.79 | 0.68 | ✓ |
| Rosaceae | *Prunus spinosa* | Blackthorn | 13.01 | - | ✓ |
| Salicaceae | *Salix* spp. | Willow | 12.54 | 0.05 | ✓ |
| Brassicaceae | *Brassica* spp. | Cabbage | 10.60 | - |  |
| Ranunculaceae | *Ranunculus ficaria* | Lesser Celandine | 8.91 | - | ✓ |
| Sapindaceae | *Acer campestre* | Field Maple | 8.22 | - | ✓ |
| Asteraceae | *Taraxacum* agg. | Dandelion | 7.11 | 21.55 | ✓ |
| Rosaceae | *Prunus* spp. | Plum (cultivated)/Cherry | 6.30 | 33.53 | ✓ |
| Plantaginaceae | *Veronica chamaedrys* | Speedwell | 4.40 | - | ✓ |
| Ranunculaceae | *Ranunculus repens* | Creeping Buttercup | 1.90 | 3.23 | ✓ |
| Brassicaceae | *Sinapsis alba* | White Mustard | 1.42 | - |  |
| Brassicaceae | *Alliaria petiolata* | Garlic Mustard | 1.02 | 0.34 | ✓ |
| Asteraceae | *Bellis perennis* | Daisy | 0.72 | 0.12 | ✓ |
| Rosaceae | *Malus* spp. | Apple / Pear | 0.71 | 13.51 | ✓ |
| Amaryllidaceae | *Narcissus pseudonarcissus* | Daffodil | 0.60 | 2.43 | ✓ |
| Brassicaceae | *Aubretia* spp. | Aubretia | 0.51 | - |  |
| Caryophyllaceae | *Stellaria media* | Common Chickweed | 0.46 | - | ✓ |
| Lamiaceae | *Glechoma hederacea* | Ground Ivy | 0.20 | Trace | ✓ |
| Lamiaceae | *Lamium album* | White Dead Nettle | 0.26 | - | ✓ |
| Apiaceae | *Anthriscus sylvestris* | Cow Parsley | 0.13 | 2.99 | ✓ |
| [Euphorbiaceae](https://en.wikipedia.org/wiki/Euphorbiaceae) | *Mercurialis perennis* | Dog's Mercury | 0.05 | - | ✓ |
| Rosaceae | *Prunus laurocerasus* | Cherry Laurel | 0.04 | - |  |
| Asteraceae | *Doronicum pardalianches* | Leopard’s Bane | 0.02 | - |  |
| Asteraceae | *Senecio vulgaris* | Groundsel | 0.01 | Trace | ✓ |
| Caryophyllaceae | *Silene dioica* | Red Campion | Trace | 0.84 | ✓ |
| Fabaceae | *Trifolium repens* | White Clover | Trace | 2.62 | ✓ |
| Asteraceae | *Tussliago farfara* | Colt’s Foot | Trace |  |  |
| Sapindaceae | *Acer pseudoplatanus* | Sycamore | - | 17.33 | ✓ |
| Rosaceae | *Filipendula ulmaria* | Meadowsweet | - | 0.47 | ✓ |
| Fagaceae | *Quercus robur* | Oak | - | 0.19 | ✓ |
| Betulaceae | *Betula* spp. | Birch | - | 0.08 | ✓ |
| Fagaceae | *Fagus sylvatica* | Beech | Trace |  |  |
| Lamiaceae | *Lamium purpureum* | Purple Dead Nettle | Trace |  | ✓ |
| Pinaceae | *Pinus sylvestris* | Scot’s Pine | Trace | Trace | ✓ |
| Rosaceae | *Potentilla* spp. | Tormentil | Trace |  |  |
| Aquifoliales | *Ilex aquifolium* | Holly |  | Trace | ✓ |
| Asteraceae | *Anthemis spp* | Mayweed |  | Trace | ✓ |
| Boraginaceae | *Symphytum officinale* | Comfrey |  | Trace | ✓ |
| Caprifoliaceae | *Sambucus nigra* | Elder |  | Trace | ✓ |
| Caryophyllaceae | *Stellaria holostea* | Greater Stitchwort |  | Trace | ✓ |
| Cornaceae | *Cornus sanguinea* | Dogwood |  | Trace | ✓ |
| Ericaceae | *Erica cineraea* | Heather |  | Trace | ✓ |
| Onagraceae | *Epilobium* spp. | Willowherb |  | Trace | ✓ |
| Plantaginaceae | *Plantago lanceolata* | Plantain |  | Trace | ✓ |
| Polygonaceae | *Rumex acetosella* | Sorrel |  | Trace | ✓ |
| Primulacaea | *Primula vulgaris* | Primrose |  | Trace | ✓ |
| Ranunculaceae | *Ranunculus acris* | Meadow Buttercup |  | Trace | ✓ |
| Sapindaceae | *Aesculus hippocastanum* | Horse Chestnut |  | Trace | ✓ |
| Violaceae | *Viola riviniana* | Dog’s Violet |  | Trace | ✓ |
|  | Unknown |  | 0.06 | 0.05 |  |
