# Supplementary material for: A bee’s-eye view of landscape change: differences in diet of 2 Andrena species (Hymenoptera: Andrenidae) between 1943 and 2021
Source: J Insect Sci. 2024 Sep 30;24(4):27. doi: 10.1093/jisesa/ieae093 (PMC11441578; doi:10.1093/jisesa/ieae093)
Supplement: ieae093_suppl_Supplementary_Table_S4 [file ieae093_suppl_supplementary_table_s4.docx]

Supp. Table S4

## Pollens in diet of *Andrena barbilabris* and flowering plants found historically and during botanical survey at Site 2 April-June 2021

| *Family* | *Species* | Common Name | Total proportion of pollen in 1943/44 samples | Total proportion of pollen in 2021 samples | Observed during 2021 botanical survey |
| --- | --- | --- | --- | --- | --- |
| Rosaceae | *Crataegus monogyna* | Hawthorn | 19.60 |  | ✓ |
| Rosaceae | *Rosa* spp. | Rose | 19.55 | 3.05 | ✓ |
| Sapindaceae | *Acer pseudoplatanus* | Sycamore | 16.09 | 0.07 | ✓ |
| Cornaceae | *Cornus* spp. | Dogwood | 14.88 | 40.69 | ✓* |
| Brassicaceae | *Sinapsis alba* | White Mustard | 7.63 |  |  |
| Apiaceae | *Chaerophyllum temulum* | Rough Chervil | 3.56 |  |  |
| Apiaceae | *Anthriscus sylvestris* | Cow Parsley | 3.53 | Trace |  |
| Rosaceae | *Rubus* spp. | Blackberry | 3.00 |  | ✓ |
| Brassicaceae | *Brassica* spp. | Brassica | 2.54 | 9.95 | ✓* |
| Salicaceae | *Salix* spp. | Willow | 1.63 |  | ✓ |
| Apiaceae | *Daucus carota* | Wild Carrot | 1.28 |  |  |
| Brassicaceae | *Barbarea vulgaris* | Winter Cress | 1.09 |  |  |
| Ranunculaceae | *Ranunculus acris* | Meadow Buttercup | 0.73 | Trace | ✓ |
| Plantaginaceae | *Veronica chamaedrys* | Speedwell | 0.73 |  |  |
| Apiaceae | *Pastinaca sativa* | Parsnip | 0.57 |  |  |
| Apiaceae | *Aegopodium podagraria* | Ground Elder | 0.39 |  |  |
| Apiaceae | *Heracleum sphondylium* | Hogweed | 0.18 |  |  |
| Asteraceae | *Leucanthemum vulgare* | Ox-eye Daisy | 0.16 |  |  |
| Asteraceae | *Anthemis arvensis* | Mayweed | 0.15 |  |  |
| Caryophyllaceae | *Silene dioica* | Red Campion | 0.09 | Trace | ✓ |
| Asteraceae | *Hypochaeris radicata* | Cat's Ear | 0.08 |  | ✓ |
| Asteraceae | *Bellis perennis* | Daisy | 0.07 | Trace | ✓ |
| Asteraceae | *Taraxacum* agg. | Dandelion | 0.04 | Trace | ✓ |
| Aquifoliales | *Ilex aquifolium* | Holly | 0.03 | 0.06 | ✓ |
| Fagaceae | *Quercus robur* | Oak | 0.02 | 0.29 | ✓ |
| Pinaceae | *Pinus sylvestris* | Scot’s Pine | 0.01 | 0.08 | ✓ |
| Polygonaceae | *Rumex acetosella* | Sorrel | 0.01 |  | ✓ |
| Asteraceae | *Senecio jacobaea* | Ragwort | 0.01 | Trace | ✓ |
| Fabaceae | *Cytisus scoparia* | Broom |  | 33.52 | ✓ |
| Rosaceae | *Prunus* spp. | Flowering Cherry | Trace | 8.53 | ✓* |
| Fagaceae | *Castanea sativa* | Sweet Chestnut |  | 3.06 | ✓* |
| Rosaceae | *Filipendula ulmaria* | Meadowsweet |  | 0.26 |  |
| Fabaceae | *Trifolium repens* | White Clover |  | 0.05 | ✓ |
| Betulaceae | *Betula* spp. | Birch | Trace | Trace | ✓ |
| Cucurbitaceae | *Bryonia alba* | White Bryony | Trace |  |  |
| Ericaceae | *Erica cinerea* | Erica | Trace | Trace | ✓ |
| Ericaceae | *Vaccinium* spp. | Bilberry | Trace |  |  |
| Fabaceae | *Vicia faba* | Broad Bean | Trace |  |  |
| Malvaceae | *Tilia* spp. | Lime | Trace |  |  |
| Plantaginaceae | *Linaria vulgaris* | Toadflax | Trace |  |  |
| Asteraceae | *Knautia* spp. | Scabious |  | Trace |  |
| Boraginaceae | *Myosotis sylvatica* | Forget-me-not |  | Trace | ✓ |
| Convolvulaceae | *Convolvulus arvensis* | Field Bindweed |  | Trace | ✓ |
| Ericaceae | *Rhododendron ponticum* | Rhododendron |  | Trace | ✓ |
| Fagaceae | *Fagus sylvatica* | Beech |  | Trace | ✓ |
| Liliaceae | *Lilium* spp. | Lily |  | Trace |  |
| Papaveraceae | *Pseudofumaria lutea* | Yellow Corydalis |  | Trace | ✓ |
| Plantaginaceae | *Plantago lanceolata* | Plantain |  | Trace | ✓ |
|  | Unknown |  | 2.34 | 0.39 |  |

*Observed more than 500 meters from nesting aggregation
